# The impact of a stress management intervention including cultural components on stress biomarker levels and mental health indicators among indigenous women

**DOI:** 10.1007/s10865-023-00391-0

**Published:** 2023-01-18

**Authors:** Amira Aker, Lena Serghides, Jasmine Cotnam, Randy Jackson, Margaret Robinson, Holly Gauvin, Christopher Mushquash, Dionne Gesink, Marni Amirault, Anita C. Benoit

**Affiliations:** 1 Department of Health & Society, University of Toronto Scarborough, Toronto, ON Canada; 2 Toronto General Hospital Research Institute, University Health Network, Toronto, Canada; 3 Department of Immunology, Institute of Medical Sciences, University of Toronto, Toronto, ON Canada; 4 Women’s College Hospital, Women’s College Research Institute, University of Toronto, Toronto, ON Canada; 5 McMaster Indigenous Research Institute, McMaster University, Hamilton, ON Canada; 6 Department of Sociology and Social Anthropology, Dalhousie University, Nova Scotia, Canada; 7 Elevate NWO, Thunder Bay, ON Canada; 8 Centre for Rural and Northern Health Research, Lakehead University, Thunder Bay, ON Canada; 9 Department of Psychology, Northern Ontario School of Medicine, Lakehead University, Thunder Bay, ON Canada; 10 Dilico Anishinabek Family Care, Fort William First Nation, Thunder Bay, ON Canada; 11 Dalla Lana School of Public Health, University of Toronto, Toronto, ON Canada; 12 Canadian Aboriginal AIDS Network, Dartmouth, NS Canada

**Keywords:** Stress, Intervention, Indigenous, Cortisol, Psychosocial intervention

## Abstract

We examined the effectiveness of a 26-week culture-inclusive intervention on reducing salivary stress biomarker levels, and perceived stress, depressive, and post-traumatic stress disorder (PTSD) symptoms measured using scales in 53 Indigenous women in Ontario, Canada. Statistical analyses compared the average biomarker levels, and the area under the curve (AUC) of biomarkers. Differences in biomarkers and mental health scale scores pre- and post-intervention were compared using mixed models with a random intercept. Interaction terms were included between the intervention and age, education, disability, and HIV status, individually, to test for sub-group differences. Cortisol AUC post-intervention was decreased compared to pre-intervention (β -1.29 µg/dL; 95%CI -2.35, -0.23). There was a slight decrease in perceived stress levels (aOR: -2.80; 95%CI -5.09, -0.50). The associations were stronger among women of younger age, higher education, and no disabilities. These interventions can be effective, but future interventions should target Indigenous population sub-groups to address individual needs.

## Background

Systemic discrimination stemming from ongoing colonialism and current trauma as well as from government-led assimilation practices, residential schools, and appropriation of land are among the most prominent determinants of disease inequities and health discrepancies in Indigenous groups across Canada (Gracey & King, 2009; King et al., 2009; Phillips-Beck et al., 2019). Despite these challenges, they have built resilience and healing in their communities (Kirmayer et al., 2003; Spicer et al., 2012). Indigenous methods of healing comprising spiritual and subsistence activities help make sense of the suffering to heal the individual (Kirmayer et al., 2003) and cultural connection is, in itself, healing at the individual and community level (Redvers & Blondin, 2020). As such, Indigenous ways of knowing and doing are essential to health interventions for Indigenous peoples.

Given that chronic stress is an immediate consequence of systemic oppression and discrimination (Williams, 2018), cognitive-behavioural stress management interventions (CBSM) and other psychosocial interventions in Indigenous settings may further build strength and resilience along with cultural approaches to healing. Psychosocial and psychological stress increases cortisol levels a product of the hypothalamic-pituitary-adrenal (HPA) axis (Adam et al., 2017; Laufer et al., 2018), and chronic stress with heightened cortisol has been implicated in the development and/or progression of mental illness (Horowitz & Zunszain, 2015) and other chronic diseases (Liu et al., 2019; Martinac et al., 2014; Mravec et al., 2020; Vitellius et al., 2018; Weinstein & Li, 2016). Stress-reducing interventions decreased cortisol levels and distress and improved mood and anxiety in study participants (Antoni et al., 2000; Cruess et al., 2000; Jones et al., 2014). Guided imagery and progressive muscle relaxation was also used to reduce cortisol levels in women living with HIV (Jones et al., 2014). In addition to cortisol, ﻿a-amylase has been shown to respond to psychosocial stress in a manner distinct from the responses of other stress biomarkers with a diurnal response that is the inverse of cortisol (Nater et al., 2006; Rohleder et al., 2004). Salivary α-amylase which has been linked to chronic pain, feelings of shame and depression, is an index for pathological autonomous nervous system dysregulation (Rohleder et al., 2008; Shirasaki et al., 2007; Stroud et al., 2009), and can be used as a marker of intervention effectiveness (Laufer et al., 2018).

CBSM, which includes cognitive behavioural skill, educational and relaxation components, has been successfully implemented in various ethnic and socioeconomic groups (Antoni et al., 2008; Jensen et al., 2013; Lopez et al., 2013; Penedo et al., 2018; Urizar et al., 2019). Despite this, there are few published studies on interventions aimed at reducing cortisol and stress among Indigenous peoples. One study assessed cortisol awakening responses (i.e., diurnal profiles) among First Nations youth (n = 11) with a laboratory induced stress response compared to non-Indigenous matched participants (n = 11) from Australia (Berger et al., 2017). Indigenous participants had a blunted cortisol awakening response associated with chronic stress experiences; however, no differences were noted in the laboratory-induced response between Indigenous and non-Indigenous participants. Differences in cortisol awakening response suggest different basal levels of cortisol among the two study groups. For example, self-reported racial discrimination was also associated with a flattened response. Flattened diurnal responses have been associated with more severe mental health conditions (Berger et al., 2017; Dedovic & Ngiam, 2015). Again, in Australia, hair cortisol and cortisone markers of chronic stress were different among Indigenous and non-Indigenous youth (Davison et al., 2019). A similar study measuring hair cortisol among First Nations people (n = 55) in Canada was higher compared to white controls (n = 32) which suggested elevated levels of chronic stress among First Nations participants (Henley et al., 2013).

Effectiveness may differ according to community sub-groups due to varying levels of external stress factors shaped by historical and ongoing colonial processes. Indigenous women in Canada are less likely to hold a postsecondary degree and be employed, and more likely to live in crowded houses requiring major repairs, compared to non-Indigenous women (Arriagada, 2016). Fewer Indigenous women living off-reserve rated their health as excellent or very good (48%) compared to non-Indigenous women (64%) and Indigenous men (53%) (Arriagada, 2016). In a study evaluating determinants of health and housing-related characteristics of people living with HIV in Ontario, Indigenous persons were more likely to be female or trans-women, be younger, have lower educational attainment, be unemployed, and be homeless or unstably housed compared to white individuals (Hawkins et al., 2009). The intersection of oppression shaped by race, gender, and class (Hancock, 2007) could vary intervention effectiveness.

Given that cultural continuity is a determinant for Indigenous People’s health (Auger, 2016), we aimed to implement a stress-reducing intervention including cultural components for Indigenous women with and without HIV in Ontario. Salivary cortisol and α-amylase levels, and scores suggestive of depressive, perceived stress and post-traumatic stress disorder symptoms were compared pre- and post-intervention to test intervention effectiveness in a single-arm trial. For our primary hypothesis, we hypothesize that the intervention will reduce stress biomarker levels and improve the scores of scales measuring perceived stress, depression and post-traumatic stress disorder. We also examined the modifying effect of sociodemographic factors and underlying morbidities on intervention effectiveness. Our secondary hypothesis states that the intervention will be less effective among women with additional external stressors.

## Materials and methods

### Study design

Our study reflects the last phase of a three-phase study called the Indigenous Women’s Stress Study (IWSS) delivered in Toronto and Thunder Bay, Ontario. IWSS aimed to identify and understand the stressors of Indigenous women, and to learn how they manage, desire to manage, adapt to, and modify stressors. All phases followed community-based research (CBR) principles and Indigenous methodology.

CBR was executed with community partners involved in designing and implementing the study (Israel et al., 1998). Our Thunder Bay partner is a health service centre delivering harm reduction and Indigenous-specific services that includes access to an Elder and cultural healing approaches. Our Indigenous partner in Toronto provides social support and services such as harm reduction and access to Indigenous peers and councillors. They are both co-authors of our manuscript. Half of the authors were Indigenous, and all hired staff were Indigenous from different nations.

An Indigenous methodology was implemented through the guidance of local Indigenous Knowledge Carriers. They also conducted ceremonies, discussed how to create a safe space and collect information from study participants using sharing circles and one-on-one discussions (Kovach, 2010). During phase 1, socio-demographic factors and measures of mental health were characterized using a peer-administered questionnaire and through peer-led interviews and sharing circles. Briefly, a sharing circle is a discussion group similar to a focus group including Indigenous protocols determined by the Knowledge Carrier (Lavallée, 2009). During phase 2, we conducted sharing circles which served as a validity check ‘focus group’ to verify phase 1 findings with study participants. Also, a culturally-inclusive stress-reducing intervention informed by phase 1 study participants was piloted and their feedback was collected to identify what they liked and disliked about the intervention components. During phase 2, the intervention was overseen by an Indigenous cultural leader and Elder who guided ceremony, informed the inclusion of Indigenous culture in the stress-reducing practices, and facilitated stress reduction practices such as guiding imagery. Phase 3 involved implementing the piloted intervention from phase 2 and incorporating study participant’s feedback. Details of the intervention are described below. In this manuscript, we report our phase 3 findings from a single-arm trial including Indigenous women in a stress-reducing intervention. More specifically, we report on the stress biomarkers as well as questionnaire data on socio-demographic and health information and scores suggestive of depressive, perceived stress and post-traumatic stress disorder symptoms from validated measures.

### Study population

Study participants living with or without HIV were recruited from Toronto and Thunder Bay, Ontario, Canada using recruitment flyers posted at several Indigenous organizations including at our community partners in Toronto and Thunder Bay. The inclusion of women living with HIV was important given our community partners include people living with HIV as part of their clients. The inclusion criteria were that participants self-identify as First Nations, Inuk or Métis, be > 16 years of age, identify their sex as female (trans-women were included in the overall study, but not in the stress biomarker analysis to account for biological sex differences), and be fluent in English or French. Our participants also had adverse socioeconomic insecurity based on the definitions of food security (International Food Policy Research Institute, 2022), housing need status (Canada Mortagage and Housing Corporation, 2014), education as an indicator for wellbeing (University of Waterloo, 2015), or the job security index and satisfaction (Probst, 2003). Women with menstrual cycle cessation were excluded due to variable concentrations of stress biomarker levels such as two to four fold increases in cortisol during pregnancy, increased variability in the circadian pattern as women age, and dysregulation of cortisol production in the case of polycystic ovarian syndrome (Burger, 2008; Hale & Burger, 2009; Woods et al., 2009, 2014).

### Intervention

An intervention session consisted of 3 components (relaxation technique, cultural or educational component, relaxation technique) developed with Indigenous facilitators and study participants’ feedback. The first component was a 15-minute relaxation technique, autogenic training, consisting of repeating a set of visualization statements focused on a sense of feeling calm, warm, or heavy arms, heart, breathing, abdomen, and forehead. The second component included 1-hour sessions of Indigenous practices (e.g., ancestral songs and dancing, drumming) to contribute to cultural continuity and educational sessions (e.g., self-regulation, stress management). The order of the sessions in the 2nd component was a cultural session followed by an educational session and so on. The third component included the second relaxation technique, a 20-45-minute guided imagery session, led by an Elder or an Indigenous healing practitioner. This component stimulated or recreated perception of sights, sounds, tastes, smells, movement and/or touch to conjure pleasant images of past or new experiences. The intervention was delivered biweekly over 26-weeks, for a total of 13 sessions and included a meal prior to beginning any activities. This timeframe was selected based on recommendations for mental health management using interventions which suggest 6 to 18 sessions with 1 to 3 weeks between sessions (Khoury & Ammar, 2014).

The second component included a first session where the study participants met each other, developed guidelines for establishing a safe space, and were gifted bundles (i.e., resources to use for ceremony such as medicines like tobacco or sweetgrass). The women were also asked about the things they would like to learn or do which informed later sessions to be specific to the needs of the women in Thunder Bay and Toronto, respectively. The topics of the sessions included: reclaiming our ancestral foods, budget conscious and culture-based nutrition, healthy sexuality and ceremonies, emotional self-regulation and attachment (led by a clinical psychologist), reclaiming ancestral languages and songs (led by a cultural facilitator), traditional dancing (led by a cultural facilitator), drumming, navigating the system (i.e., access and use of health services), and effects of stress (led by a scientist with expertise in stress biomarkers) and managing stress. The content of the remaining sessions differed between groups because they were informed by the participants and included: Four Directions Teachings (Department of Canadian Heritage, 2006–2012) and other cultural teachings, making dream catchers, painting combined with emotional regulation, learning Cree syllabics (led by a cultural facilitator), jingle dress visioning (led by a cultural facilitator), effect of stress on sleep (led by an expert on the topic), drumming, radical acceptance (led by a clinical psychologist), ask a pharmacist (led by a pharmacist) and naloxone training (led by an outreach worker). In Toronto, the intervention was facilitated by a First Nations research assistant at 3 distinct Indigenous organizations in collaboration with our community partner with and without guest speakers. An Elder participated in some sessions to discuss ceremonies and teachings. In Thunder Bay, a Métis research assistant and Elder facilitated the sessions with and without the guest speakers, and our community partner was the only study site. The Elder led the ceremonies and provided teachings. Multiple sites were necessary in Toronto, since some participants were not comfortable at the different sites and upon recruitment they were asked where they would like to attend the intervention.

The order of the sessions and start dates differed between groups. The start dates were staggered to consider the availability of cultural service providers and other topic experts as well as to accommodate the research staff. The same staff worked on each intervention in their respective cities and were at certain points in the study working with 3 (Toronto) to 4 (Thunder Bay) different groups of women in one week to plan and lead sessions as well as collect data. Also, participants in Toronto were recruited over the following periods: May 1st – June 30th, 2017 and July 1st – August 31st, 2017. Participants in Thunder Bay were recruited over two time periods: May 1st – June 30th, 2017 and February 1st to May 31st, 2018. There were 3 intervention groups (n = 11, n = 9, n = 8) held in Toronto and 4 groups in Thunder Bay (n = 12, n = 11, n = 9, n = 10), Ontario. The sessions were held from May 2017 until March 2019 at community partner sites.

### Biomarkers

Saliva was collected at 2 time points: the baseline visit occurring 1–7 days pre-intervention and the final study visit at 1–7 days post-intervention. Five saliva samples were collected per day with the first sample collected at waking, the second 30–60 min after waking, and the remaining 3 samples 3–4 h apart.

Participants were provided with a saliva collection package (saliva collection tubes, lunch bag with ice packs, latex-free gloves, saliva collection checklist) and detailed sampling instructions with a descriptive flowchart (Salimetrics®, 2022). The exchange of the saliva samples was done at a location and time convenient for the study participants. Saliva samples were stored at 4^o^C for up to 12 h, after which they were transferred to the laboratory and stored at -80^o^C until analysis using Salimetrics® enzyme immunoassay kits (State College, PA). Cortisol and α-amylase concentrations were measured from the saliva (Salimetrics®, 2019, 2021). Cortisol levels had an intra-assay coefficient of variation 4–7% and an inter-assay coefficient of variation 3.0–11.0%. α-Amylase levels had an intra-assay coefficient of variation 2.5–7.2% and an inter-assay coefficient of variation 3.6–5.8%. Saliva samples with blood were excluded (N = 5). One more sample was dropped for having extreme (> 3rd quartile + 3*(interquartile range), influential values.

The daily average concentration for each biomarker was defined by taking a mean of the logged biomarker concentrations across a day for every study participant at pre- and post- intervention. The total daily secretion of cortisol and α-amylase were calculated using the area under the curve with respect to the ground (AUC_g_) with the trapezoid rule area for each participant at pre- and post-intervention.

### Mental health

Our study participants reported high levels of over the counter and prescribed medication use that may impact their cortisol and α-amylase concentrations. As such, mental health outcomes, perceived stress, depressive, and post-traumatic stress disorder symptoms were measured as additional outcomes of interest to test the effectiveness of the intervention. Perceived stress was measured using the 10-item Perceived Stress Scale (PSS) which has shown good validity and reliability (Cohen et al., 1983), and internal consistency in Indigenous females (coefficient alpha = 0.82) (Benoit et al., 2016). PSS is a self-report scale of feelings and thoughts regarding a loss of control and lack of predictability with answer options ranging from 0 (never) to 4 (very often). Scores > 13 were considered low/average stress, scores 13–20 high stress levels, and scores > 20 chronic stress. Depressive symptoms were measured with the 20-item Center for Epidemiological Studies Depression Scale (CES-D) (Radloff, 1977). CES-D is a widely-used tool with good internal reliability (Anderson et al., 2011) and internal consistency in Indigenous females (coefficient alpha = 0.89) (Benoit et al., 2016). It is a self-report measure on how participants felt or behaved during the past week on a scale of 1 (rarely or none of the time) to 4 (most or all of the time). Scores ≥ 16 are suggestive of significant depressive symptoms, and scores greater > 21 of severe symptoms. Traumatic and stressful life experiences were measured using the Post Traumatic Stress Disorder Checklist-Civilian Version (PCL-C) (Weathers et al., 1994). The self-report scale has good reliability and internal validity (Conybeare et al., 2012; Weathers et al., 1993), and good internal consistency among Indigenous females (coefficient alpha = 0.93) (Benoit et al., 2016). The scale had responses ranging from 1 (not at all) to 5 (extremely). Scores > 44 were considered clinically significant.

### Other variables

Participants completed a form indicating if they had eaten, smoked cigarettes, consumed alcohol or marijuana, performed a physical activity, or had a stressful event prior to taking a saliva sample. They recorded their mood level at the time of sampling (1: Great − 5: Bad). These variables were included in the main biomarker models as covariates since they could impact biomarker levels (Adam & Kumari, 2009).

Participants also completed a questionnaire on sociodemographic and morbidity characteristics pre- and post-intervention. These included age, education, income, Indigenous group, disability status, and HIV status. The sociodemographic variables (age and education level) and morbidity status (disability and HIV status) were selected for effect modification analysis. Age is an important consideration of biomarker levels and mental health status (Yiallouris et al., 2019). Two age categories were created with 45 years as the cut-off based on suggestions by an endocrinologist to consider possible fluctuations in the menstrual cycle because of pre-menopause and in women living with HIV early or premature menopause has been reported in their mid-40s (Andany et al., 2016). This cut-off also enabled similar sample sizes between the two groups for analysis purposes. Education level was a proxy for socioeconomic status because there was less variation in income level in our study population. Additionally, there was a significant prevalence of disability in our population, and it was included as a potential effect modifier. People living with HIV are reported to be at higher risk for mental illnesses (Cain et al., 2013; Remien et al., 2019; X); therefore, HIV status was also included in interaction analyses.

### Statistical analyses

Summary demographics were compared using proportions in the full dataset. Differences in pre-intervention cortisol and α-amylase concentrations were compared across participant characteristics using linear models. For the analyses comparing stress biomarkers pre- and post-intervention, three main models were constructed. The first analyzed daily average concentrations of cortisol and α-amylase pre- and post-intervention using a paired t-test. The second analyzed the total daily secretion of cortisol and α-amylase using a paired t-test. Lastly, a mixed effect model with a random intercept was conducted to test the effect of the intervention on logged cortisol and α-amylase concentrations (due to their non-normal distributions). The estimated parameters from the models were used to calculate the AUC pre- and post-intervention to account for group summary statistics rather than individual summary measures. The mixed effect model was superior to individual AUC measures in the case of missing data (Bell et al., 2014). Unadjusted models included the intervention group and time variables. Adjusted models included covariates that changed the main effect estimate by ≥ 10%. A quadratic time variable was included as a potential covariate to account for non-linearity. The new AUC measures were compared using a t-test. We hypothesized a decrease in the daily averages and total daily secretions of cortisol and α-amylase post-intervention compared to pre-intervention. Interaction terms between the intervention group and age, education, disability status, and HIV status were each added in the model to test for effect modification.

For the analyses comparing mental health pre- and post-intervention, we used the PSS, CES-D, and PCL-C as continuous and categorical variables. The advantage of the continuous variables was additional power, whereas the categorical variables allowed the use of cut-off values. Linear mixed effect models with a random intercept were constructed for each of the continuous measures. Modified Poisson regression models were constructed for each of the categorical scales to allow for repeated subject measures. CES-D and PSS models used a cumulative logit distribution to account for the three outcome levels (CES-D: <16, 16–21, > 21; PSS: <13, 13–20, > 20), and the PCL-C model used a binomial distribution to account for two outcome levels (< 45, ≥ 45). We hypothesized the scores of scales measuring perceived stress, depressive and post-traumatic stress disorder will be improved post-intervention compared to pre-intervention. Again, we tested for effect modification by including an interaction term between the intervention group and each of age, education, disability status, and HIV status. The type I error level was set at 5% and we conducted all analyses in SAS 9.4.

## Results

### Baseline study participant characteristics

Seventy-two women were recruited, and 34 participated in salivary stress biomarker analysis. Of the 72 women, 13 women withdrew before intervention completion. Two women with missing demographic data were excluded from our analyses. There were 57 participants aged 24–66 years, with 46% aged between 24 and 45 years (Table 1). Over half the women (65%) had up to a high school education, and 86% had a household income <$20,000. Additionally, 63% of the women reported having a disability and 12% were living with HIV.

**Table 1:** Baseline characteristics of study participants at pre-intervention and of study participants by stress biomarker level at pre-intervention

		Pre-intervention	Stress biomarker at pre-intervention**		
		N (%)	N (%)	α-Amylase (U/mL) Median (IQR)	Cortisol (µg/dL) Median (IQR)
**Age (years)**	24–45	26 (46)	13 (43)	310.9 (113.6)	42.8 (72.2)
	46–66	31 (54)	17 (57)	264.2 (79.6)*	145.5 (128.6)*
**Education**	High school or less	37 (65)	14 (47)	300.2 (79.7)	78.9 (95.0)
	Some post-secondary	20 (35)	16 (53)	274.4 (113.1)	126.1 (132.4)
**Income**	<$20,000	49 (86)	26 (87)	297.1 (96.6)	111.3 (147.4)
	$20,000 - $40,000	8 (14)	4 (13)	273.3 (40.0)	54.9 (143.9)
**Relationship**	Married/Common law	18 (32)	9 (30)	264.2 (85.7)	100.3 (82.4)
	Single	39 (68)	21 (70)	294.7 (84.0)	202.6 (195.4)
**Disability**	No disabilities	21 (37)	12 (40)	307.2 (149.2)	87.3 (108.0)
	Disabilities present	36 (63)	18 (60)	276.2 (88.8)*	111.2 (162.1)
**HIV Status**	No HIV	50 (88)	21 (70)	288.1 (86.5)	100.7 (135.8)
	Living with HIV	7 (12)	9 (30)	305.7 (50.2)	114.7 (134.8)

* P value from linear regression models regressing stress biomarker and study population characteristic at pre-intervention < 0.05

** Thirty-one participants had saliva without blood, but one participant was excluded for missing demographic data

Of the 31 women with stress biomarker data, 30 of the women also had complete demographic data. Cortisol levels were lower among older women and women with disabilities, compared to younger women and women currently without disabilities, respectively (Table 1). By contrast, α-amylase levels were higher among older women compared to younger women. Supplemental Fig. 1 provides the numbers of participants over the course of the study. The attendance rates in Toronto were 79%, 61%, 83%, and in Thunder Bay the rates were 87%, 89%, 95%, and 92%.

### Stress biomarkers

**Fig. 1:**
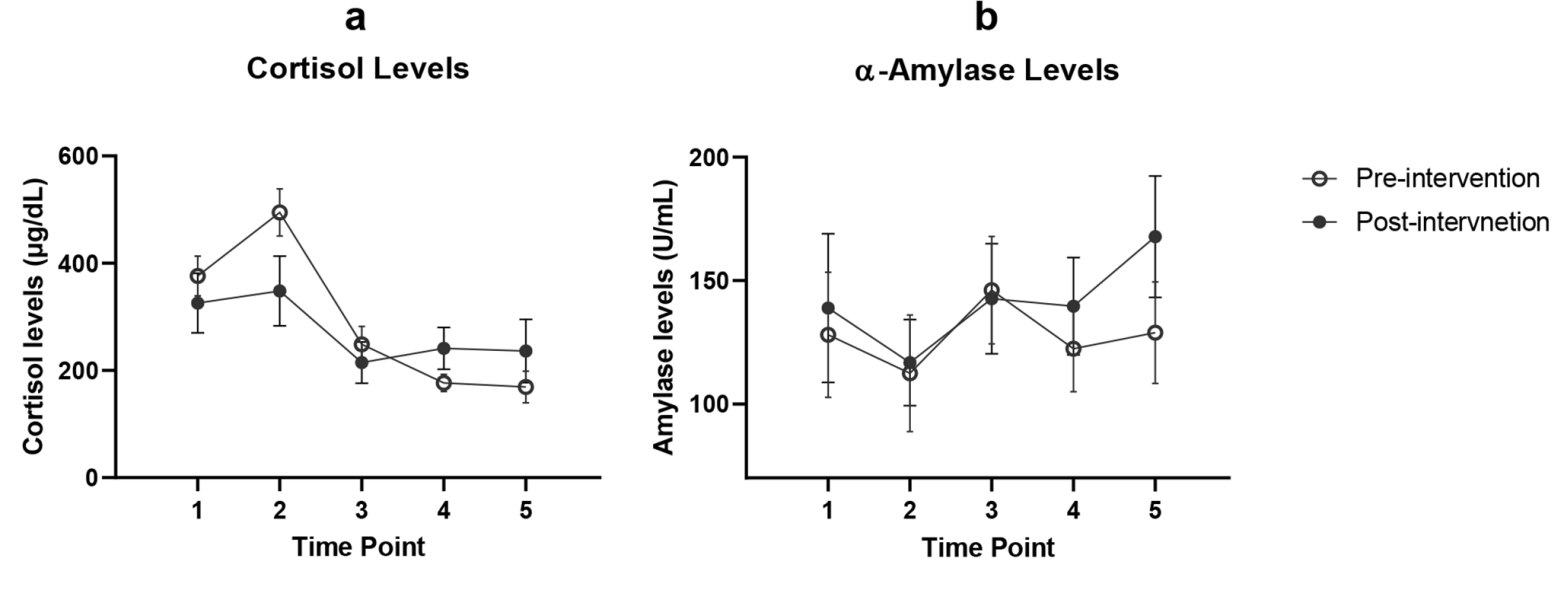
Salivary cortisol (a) and α-amylase (b) mean and standardized error of mean versus time pre-intervention (N = 31) and post-intervention (N = 19)

Cortisol levels were higher at waking compared to later in the day, with a peak at the second time point (within an hour of waking) at pre- and post-intervention (Fig. 1). α-Amylase levels showed greater variation throughout the day, with a drop at the second time point, and increases at later time points post-intervention. There was no difference in daily average cortisol (mean difference: -0.12 µg/dL; 95% CI -0.40, 0.16; p = 0.38) and daily average α-amylase (mean difference: 0.02 U/mL; 95% CI -0.28, 0.32; p = 0.87) concentrations pre- and post- intervention (Table 2).

**Table 2:** Differences between daily average concentrations of cortisol and α-amylase before and after intervention

	N	Difference (95% CI)	P value
**Cortisol (µg/dL)**	16	-0.12 (-0.40, 0.16)	0.38
**α-Amylase (U/mL)**	16	0.02 (-0.28, 0.32)	0.87

Analyses comparing daily average concentrations and AUC from individual data required complete pre- and post- intervention data.

**Fig. 2:**
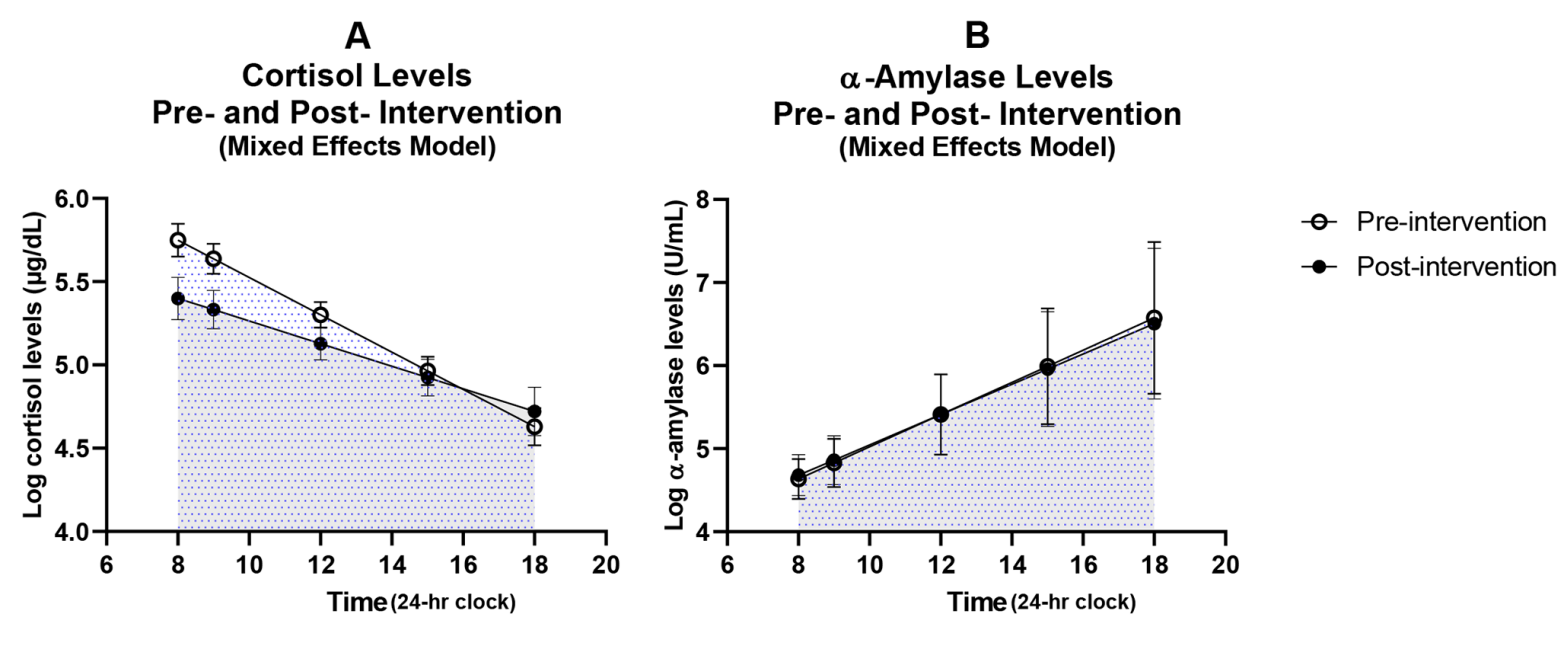
Log cortisol and α-amylase levels versus time pre- and post- intervention from mixed effect model results (cortisol N = 33; α-amylase N = 32)

The time*intervention p-value was = 0.06, indicating a marginally significant association between the intervention and a decrease in cortisol levels (Table 3a). There was no significant association between the intervention and α-amylase in mixed analyses. Similar to results of the AUC from individual data (Table 3b), cortisol AUC measures using summary statistics from mixed models was decreased post-intervention (p = 0.02), and no difference was observed in the α-amylase AUC using summary statistics (p = 1.00) (Table 3c). Figure 2 suggests that cortisol levels post-intervention decreased during earlier time points of the day.

**Table 3a:** Mixed effects model results testing difference in stress biomarker levels before and after intervention

	N	Time p value	Intervention p value	Time*Intervention p value
**Cortisol**	33	< 0.0001	0.02	0.06
**α-Amylase**	32	0.01	0.63	0.60

The final cortisol model adjusted for recreational exercise before taking a sample.

The final amylase model adjusted for experiencing a stressful event or eating a meal prior to taking a sample and included a quadratic term for time to account for non-linearity.

**Table 3b:** Differences between the total daily secretion (AUC) of cortisol and α-amylase from individual raw data before and after intervention

	N	Difference (95% CI)	P value
**Cortisol**	16	-340.1 (-631.7, -48.6)	0.03
**α-Amylase**	16	-8.5 (-164.0, 147.1)	0.90

Analyses comparing daily average concentrations and AUC from individual data required complete pre- and post- intervention data.

**Table 3c:** Differences between the AUC of stress biomarkers from mixed effect models before and after intervention

	N	Difference (95% CI)	P value
**Cortisol**	33	-1.29 (-2.35, -0.23)	0.02
**α -Amylase**	32	-0.12 (-99.35, 99.11)	1.00

The final cortisol model adjusted for recreational exercise before taking a sample.

The final amylase model adjusted for experiencing a stressful event or eating a meal prior to taking a sample and included a quadratic term for time to account for non-linearity.

**Fig. 3:**
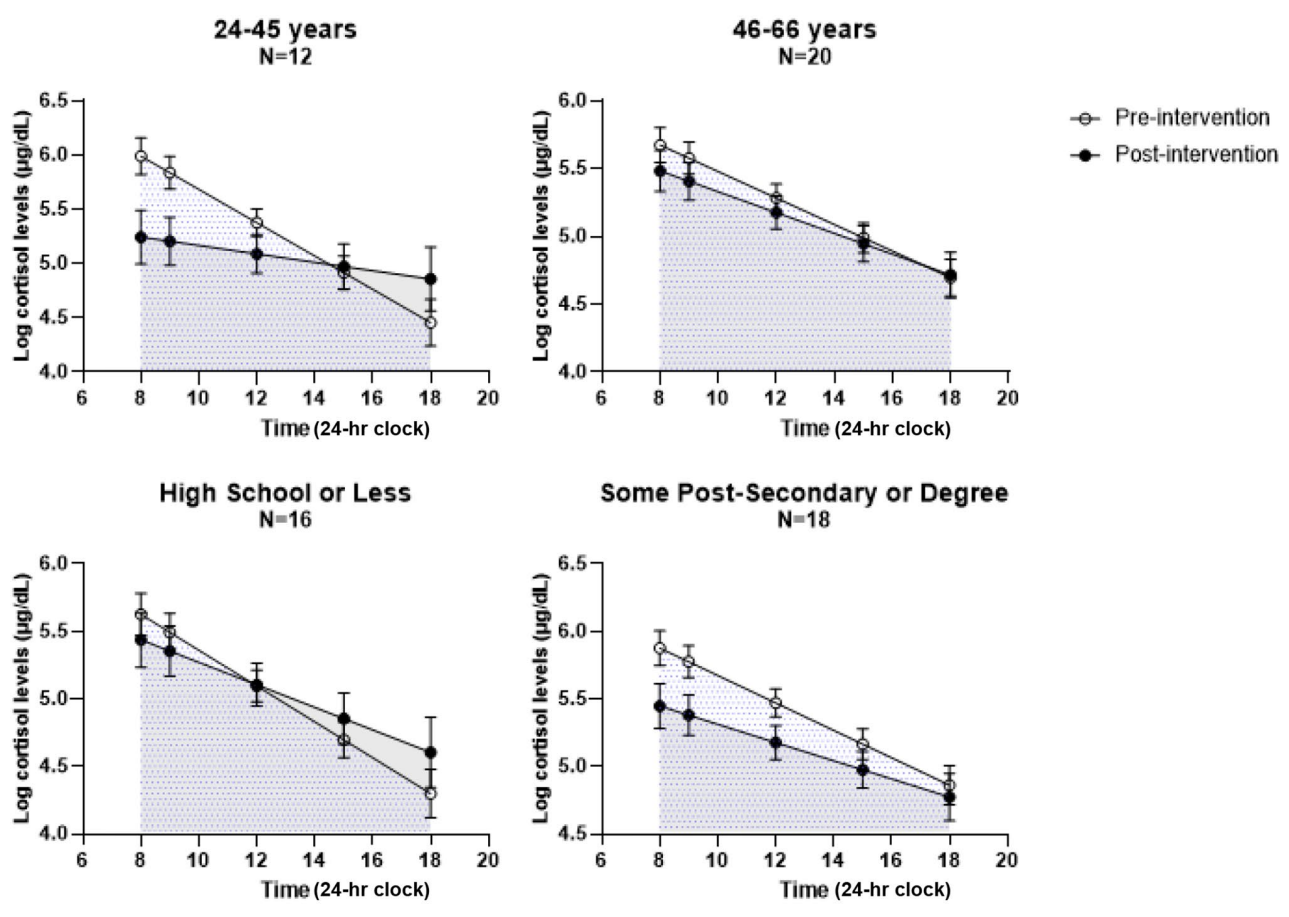
Stratified cortisol concentrations versus time pre- and post-intervention by age and education. Interaction p-value for age = 0.17 and for education p = 0.05

**Fig. 4:**
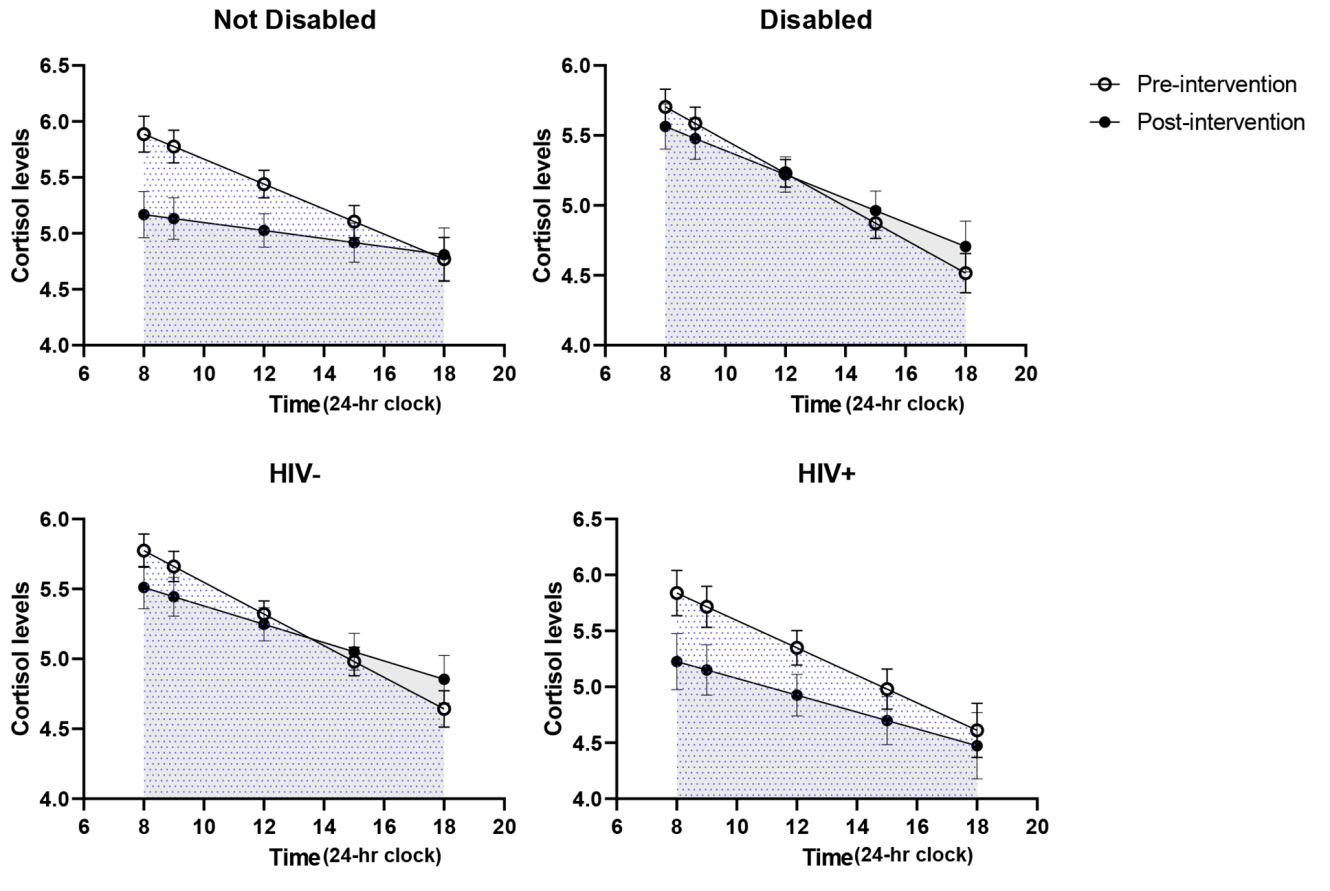
Stratified cortisol concentrations versus time pre- and post-intervention by disability and HIV status. Interaction p-value for disability = 0.02 and for HIV p = 0.12

There was a decrease in cortisol levels post-intervention among women with at least some postsecondary education (interaction p = 0.05) and no disabilities (interaction p = 0.02), but not among women with ≤ high school (interaction p = 0.17) or disabilities (interaction p = 0.12) (Figs. 3 and 4). Although not statistically significant, there was a decrease in cortisol levels post-intervention among women with HIV, and a smaller decrease among women without HIV (Fig. 4). Cortisol levels among women aged 24–45 and with no disabilities had a flattened curve post-intervention.

### Psycho-social measures

**Fig. 5:**
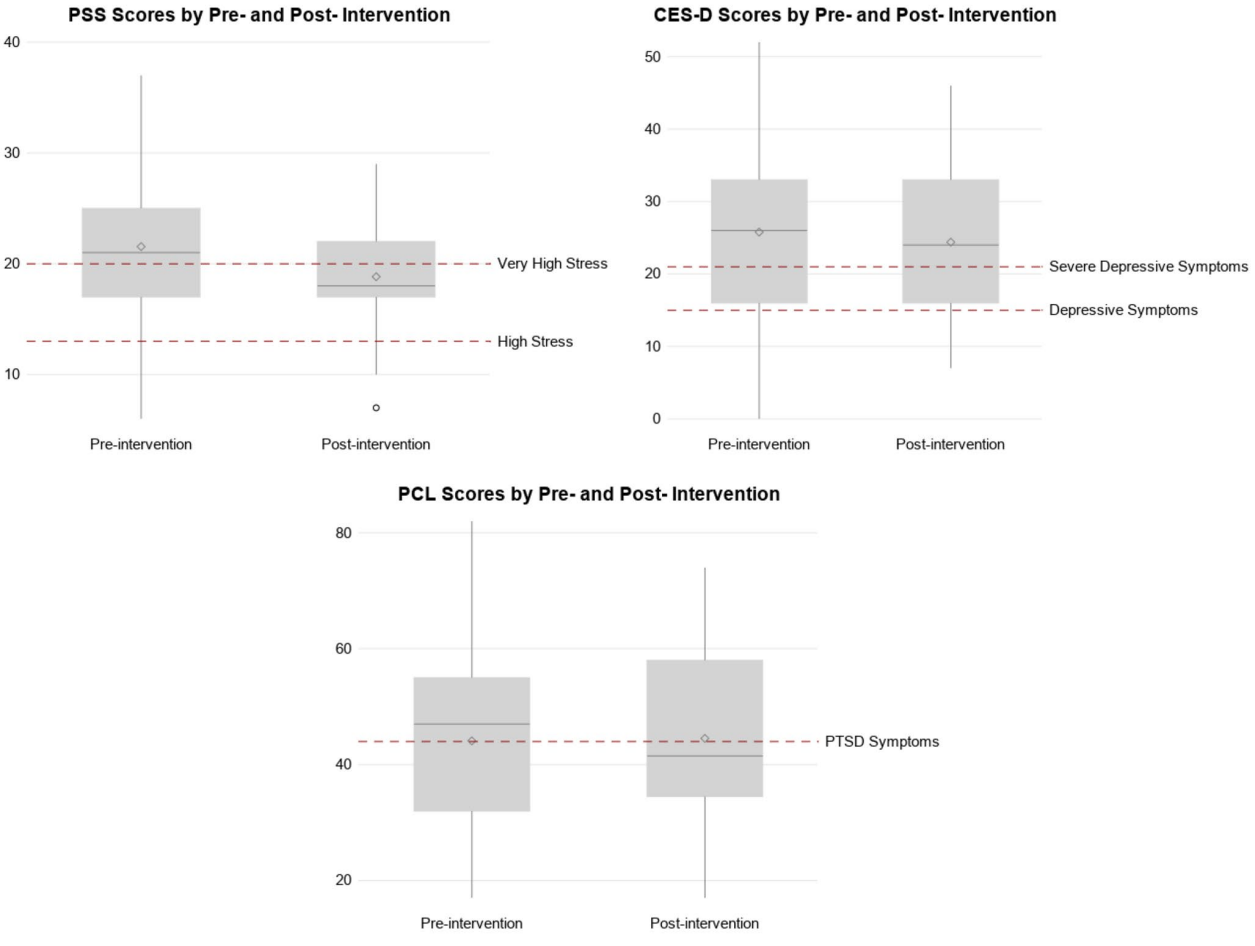
Psychosocial scale scores (PSS, CES-D, and PCL) by pre- and post- intervention for the 53 study participants. Cut-off values for categorical variables denoted by red lines

There was a slight decrease in perceived stress (PSS), depression (CES-D), and post-traumatic stress disorder (PCL-C) scores after the intervention, but only the decrease in PSS was significant (aβ -2.80; 95% CI -5.09, -0.50) (Table 4a & Fig. 5). Categorical scores of PSS (high and moderate perceived stress symptoms) and PCL (PTSD symptoms) decreased post-intervention (aOR: 0.50; 95% CI 0.25, 1.00, and aOR:0.51; 95% CI 0.26, 1.00, respectively) (Table 4b). The intervention was not associated with depressive symptoms (aOR: 0.63; 95% CI 0.32, 1.26), as compared to no symptoms. Younger women had greater reductions in symptoms of depression and PTSD (p value = 0.02 and 0.03) (Additional Files 1–3). There was no evidence of effect modification of scores by any other factors (education, disability, or HIV status).

**Table 4a:** Difference in continuous psychosocial scores pre- and post-intervention

	N	Unadjusted β (95% CI)	Adjusted β (95% CI)
**PSS**	53	-2.59 (-4.75, -0.43)	-2.80 (-5.09, -0.50)
**CES-D**	53	-3.42 (-7.30, 0.46)	-3.58 (-7.64, 0.47)
**PCL**	53	-2.67 (-8.22, 2.88)	-2.99 (-8.90, 2.92)

Final models adjusted for age, education, relationship status, disability status, and HIV status.

**Table 4b:** Difference in categorical psychosocial scores pre- and post-intervention

	N	Unadjusted OR (95% CI)	Adjusted OR (95% CI)
**PSS**	53	0.50 (0.25, 1.01)	0.50 (0.25, 1.00)
**CES-D**	53	0.63 (0.31, 1.27)	0.63 (0.32, 1.26)
**PCL**	53	0.46 (0.24, 0.89)*	0.51 (0.26, 1.00)*

Final models adjusted for age, education, relationship status, disability status, and HIV status.

* p-value < 0.05

## Discussion

After implementing a stress-reducing intervention including culture for Indigenous women with or without HIV, we observed reduced salivary cortisol levels after the intervention but no change in α-amylase levels, and a slight decrease in perceived stress. Decreased cortisol levels were more apparent among women with a higher educational attainment and those without disabilities; both were variables suggestive of fewer socio-economic challenges. Decreases in depressive and PTSD scores was more apparent in younger women. Our results indicate that culturally inclusive stress-reducing interventions may be an effective tool to reduce chronic stress in Indigenous populations, though this one intervention does not reduce the continuing need for broad systemic changes to address the origins of stress.

Our results with respect to cortisol and the mental health scales are aligned with other studies, where psychosocial interventions have been effective at reducing stress in other populations, including among women with breast cancer (Tang et al., 2020), depression (Abbasian et al., 2014), HIV (Brown & Vanable, 2008; McIntosh et al., 2019), during pregnancy (Urizar et al., 2019), and specifically Indigenous communities (Berger et al., 2017). For example, there was a significant decrease in cortisol output (calculated using AUC) among 47 women at risk for breast cancer in a CBSM intervention group as compared to 44 women in a control group (Sannes et al., 2015). In a randomized control study of 158 women at high risk for breast cancer, intervention participants reported lower depressive symptoms and perceived stress as compared to controls (McGregor et al., 2015). We did not observe a change in depressive symptoms but did observe a decrease in perceived stress and PTSD symptoms. Relaxation plays a major role in CBSM interventions and appears to be the key to reducing stress. One study provided recordings of relaxation exercises to encourage daily practice which increased the ability to relax, and reduced tension, depression, and overall negative mood (Antoni et al., 2006), likely due to a decrease in cortisol levels (Phillips et al., 2008).We did, however, observe an unexpected result of flatter diurnal cortisol slopes post-intervention. Flatter slopes are usually indicative of chronic stress, unfavourable biologic changes, and poorer physiological functioning (Adam et al., 2017), and may indicate the presence of severe mental health conditions (Berger et al., 2017; Dedovic & Ngiam, 2015). However, our findings may also be reflective of our small sample size or of the increased variability in results pre-intervention due to several women withdrawing from our study.

We observed a stronger impact of the intervention on the HPA axis (via cortisol) over the sympathetic nervous system (via α-amylase). Our finding with regards to α-amylase was in line with those of a study examining salivary cortisol and α-amylase in German Armed Forces service members accessing an internet-based trauma-focused cognitive behavioral therapy (Schumacher et al., 2022). The study reported no differences in either cortisol or α-amylase after the intervention; however, participants with PTSD had flatter cortisol slopes and lower daily output. Alternatively, therapeutic changes in biological/hormonal systems can be delayed (Laufer et al., 2018), and our study may have been unable to test for such changes.

This is the first study to examine the effectiveness of a stress-reducing intervention including culture by participant characteristics. We observed greater reductions in cortisol levels in women with higher educational attainments and those without disabilities, and greater reductions in depressive and PTSD scores among younger women. This could indicate that the intervention was less effective among women with potentially greater external stress-inducing factors, including lower educational status, disability, and older age. Similar externalizing difficulties faced by many Indigenous groups have been highlighted in several studies to inform mental health and therapeutic interventions (Garrett et al., 2014). This suggests a need for more targeted strategies to address sub-group needs. Alternatively, community-based interventions may be a more effective tool in reducing chronic stress by stimulating further cultural continuity and connection (Auger, 2016; Kirmayer et al., 2003). Mental health promotion could benefit from a basis in collective identity, community engagement, and empowerment to re-identify and strengthen individual roles within a community (Garrett et al., 2014; Kirmayer et al., 2003). Future studies could combine community and individual-based interventions to build community resilience and strength, while simultaneously targeting sub-groups for tailored interventions. However, while these interventions help build resilience, only eliminating societal structural features that support colonialism are the real “solutions”.

Our study had several strengths, including the administration of an intervention inclusive of culture, community leadership in the implementation and delivery of the intervention, high attendance rates, analysis of both stress biomarkers (markers for the HPA axis and autonomic nervous system) and mental health scales. Some limitations were our small sample size, the relatively high drop-out rate, and lack of a control group to compare the effect of the intervention. The study population experienced many life fluctuations that contributed to the high attrition rate. We were also unable to assess the influence of adverse childhood experiences or medication use on our intervention effect (Gilgoff et al., 2020). There is a need for future studies to identify the key elements of a stress-reducing program yielding the best results, and to compare the results to a control group to assess which cultural components may be more effective in reducing stress (Clifford & Shakeshaft, 2017; Clifford et al., 2013; Harlow et al., 2014). Increased measures for follow-up would also be beneficial to understand the long-term effects of an intervention. An increased sampling of stress biomarkers throughout the day would better capture biomarker hourly fluctuations and intraindividual variability for further analyses. Given the self-reported nature of the protocol adherence, it is difficult to accurately ascertain how adherent participants were with saliva collection. Finally, we did not correct for multiple comparisons due to our low sample size but the concordance across our results lend confidence to our interpretations.

## Conclusion

After implementing a culture inclusive stress-reducing intervention, we observed an overall decrease in the stress biomarker cortisol, but not α-amylase levels in a select group of Indigenous women. The intervention was more effective among women who were younger and had a higher educational attainment with no disabilities. Our study provides evidence for the intervention’s effectiveness in reducing stress in a select group of Indigenous women with socio-economic insecurities. However, it is important to note that many Indigenous women experience systemic and structural barriers to achieving mental health and wellbeing that are rooted in the intermediate (e.g., racism, poverty, economic insecurity, inadequate housing) and distal determinants of health (e.g., residential school, Indian act, colonialism, political decision-making). These barriers which exist along their life course shape their proximal determinants of health (e.g., life stressors, diabetes, heart disease, mental health and addiction) which includes the experiences of the women in our study. Although our intervention was effective, a shift more than a change in how to address systemic and structural barriers are needed for reconciliation beyond being merely performative acts and gestures. One such change could include how a community partner used the intervention in this study to inform long-term programming and hired Indigenous service providers to contribute to a shift in the types of health services available to Indigenous clients.

## Data Availability

The datasets generated and/or analysed during the current study are not publicly available due to the sensitive nature of the data but are available from the corresponding author on reasonable request.
